# Comparison of Phaco-Tanito Microhook Trabeculotomy between Propensity-Score-Matched 120-Degree and 240-Degree Incision Groups

**DOI:** 10.3390/jcm12237460

**Published:** 2023-12-01

**Authors:** Kazunobu Sugihara, Ayaka Shimada, Sho Ichioka, Akiko Harano, Masaki Tanito

**Affiliations:** Department of Ophthalmology, Shimane University Faculty of Medicine, Enya 89-1, Izumo 693-8501, Japanishidaki@med.shimane-u.ac.jp (A.H.)

**Keywords:** microhook ab-interno trabeculotomy, Tanito Microhook Trabeculotomy (TMH), intraocular pressure, minimally invasive glaucoma surgery, cataract surgery, propensity score matching

## Abstract

This study compared the effectiveness and safety of 120-degree (nasal) and 240-degree (bilateral) incisions in Tanito Microhook Trabeculotomy (TMH) combined with cataract surgery in patients with open-angle glaucoma. From a pool of 185 eyes, 67 eyes from 67 subjects were selected for each incision group using propensity score matching to align age, sex, glaucoma type, and preoperative intraocular pressure (IOP). The study found that preoperative IOP, initially 18.6 mmHg in both groups, decreased to 13.2 mmHg in the nasal group and 12.8 mmHg in the bilateral group 12 months postoperatively, representing reductions of 29% and 31%, respectively. Similarly, medication scores decreased from 3.4 to 2.7 in the nasal group and from 3.1 to 2.5 in the bilateral group. Notably, the bilateral incision group exhibited a significantly higher hyphema red blood cell score compared to the nasal group (*p* < 0.0001). Across the study period, other parameters such as IOP, medication score, visual acuity, anterior chamber flare, corneal endothelial cell density, visual field mean deviation, and the frequency of surgical complications other than hyphema were similar between the groups. The study concluded that TMH combined with cataract surgery is equally effective and safe regardless of incision width, although narrower incisions resulted in reduced early postoperative hyphema.

## 1. Introduction

Glaucoma, a group of neurodegenerative ophthalmic diseases, leads to optic nerve damage, visual field constriction, and vision loss [1]. Elevated intraocular pressure (IOP) is a significant risk factor for glaucoma, and the only proven treatment involves IOP reduction through medication, laser therapy, or incisional surgery. Minimally Invasive Glaucoma Surgery (MIGS) has emerged as an effective strategy, offering favorable outcomes and enhanced safety [2]. The Tanito microhook ab interno trabeculotomy (TMH), a MIGS procedure using a metal hook to incise the trabecular meshwork has been noted to effectively reduce IOP and decrease dependency on antiglaucoma medications [3].

Originally, TMH involved incising both nasal and temporal trabecular meshworks with three types of hooks, resulting in a 240-degree incision. Studies on cadaver eyes suggested that the extent of trabeculotomy incision influences IOP reduction [4]. While some studies reported varying IOP reduction efficacy based on the width of the surgical extent [5,6,7], others found no such differences [8,9,10,11,12,13]. Assessments of the correlation between surgical extent and IOP reduction efficacy often involved comparing different surgical procedures or groups with varying preoperative IOP or patient backgrounds [5,6,7,10,13]. Some studies included only a small number of subjects [8,11]. Although the results are not conclusive [14,15,16], cataract removal by goniotomy may favorably impact IOP reduction [17]. Thus, including both combined cataract and goniotomy and goniotomy alone in efficacy assessments can introduce bias.

In the current study, we comprehensively compared surgical efficacy and safety profiles between 120-degree and 240-degree TMH to evaluate the impact of surgical extent on IOP reduction. To minimize confounding factors, the study included only patients with open-angle glaucoma undergoing combined cataract and TMH surgery performed by a single surgeon. Additionally, patient background factors such as age, sex, preoperative IOP, and glaucoma type were matched between the two groups using propensity score matching before comparison.

## 2. Subjects and Methods

### 2.1. Participants

This study adhered to the tenets of the Declaration of Helsinki and was approved by the institutional review board (IRB) of Shimane University Hospital (IRB No. 20200517-1, revised protocol issued on 15 June 2023). All participants provided written informed consent for surgery prior to the surgery. The IRB approval did not require individual written informed consent for publication. Instead, the study protocol was publicly posted at the study institutions to inform participants about the study. Only anonymized data were used for statistical analysis.

We retrospectively included participants who met the following criteria: individuals who underwent surgery performed by one surgeon (M.T.) at Shimane University Hospital from April 2018 to August 2021; those who underwent combined TMH and cataract surgery; and those diagnosed with primary open angle glaucoma (POAG) or exfoliation glaucoma (EXG), with no history of previous intraocular or glaucoma surgeries, and were followed up for at least 12 months postoperatively. The exclusion criteria included intraoperative posterior capsular rupture or Zinn’s zonular dialysis. In total, 185 eyes were selected from our institutional database. For comparison between nasal-only incision and bilateral incision procedures in TMH, an equal number of eyes from each group were selected using propensity score matching (caliper coefficient = 0.20), based on age, sex, preoperative IOP, and glaucoma type. Only one eye per subject was chosen for the matching. In our hospital, bilateral incision was predominantly used until June 2019, while nasal incision was primarily chosen from July 2019, with most cases treated with nasal incision after April 2021. This chronological change enabled us to collect data from both nasal and bilateral incisions, resulting in 67 eyes from 67 subjects being selected for each group.

### 2.2. Measurements

We collected clinical parameters from medical charts, including age, sex, glaucoma type, ocular surgical history, surgical procedure, preoperative and postoperative best corrected visual acuities (BCVA), IOP, number of antiglaucoma medications, anterior chamber (AC) flare (measured using the FM-600 laser flare meter, Kowa, Nagoya, Japan), corneal endothelial cell density (CECD) (measured using the EM-3000 specular microscope, Tomey, Nagoya, Japan), axial length (measured using the OA2000 optical biometer, Tomey), visual field MD (central 30-2 program, Humphrey Visual Field Analyzer, Carl Zeiss Meditec, Dublin, CA, USA), and the duration of postoperative follow-up. Subjects with exfoliation material in only one eye (by slit lamp examination) were defined as unilateral EXG; the other eye was defined as POAG. Decimal BCVA was converted to the logarithm of the minimum angle of resolution VA. Counting fingers, hand motions, light perception, and no light perception were assigned decimal VAs of 0.0025, 0.002, 0.0016, and 0.0013, respectively [18]. IOP was measured using Goldmann applanation tonometry, except on postoperative day 3 when the iCARE rebound tonometer (M.E. Technica, Tokyo, Japan) was used. The medication score accounted for 1 point per component of topical medication or 1 tablet of oral acetazolamide. Information on the site of trabeculotomy, perioperative complications, interventions for complications, and additional glaucoma surgeries were collected from medical and surgical records. The postoperative follow-up periods were defined as 1–3 days for post-operative day (POD) 3, 1–3 weeks for postoperative week (POW) 2, 2–4 months for post-operative month (POM) 3, 5–7 months for POM 6, 8–10 months for POM 9, and 11–14 months for POM 12.

The Shimane University Postoperative Hyphema Scoring system (SU-RLC) [19] is utilized to assess the severity of hyphema. This method evaluates hyphema severity through a slit-lamp examination, focusing on three primary elements: red blood cells (RBCs) (R), layering (L), and clot formation (C). The RBCs aspect (R) is rated on a scale from 0 (no visible floating RBCs in the Anterior Chamber [AC]) to 3 (dense floating RBCs obscuring iris patterns). The layering factor (L) is scored from 0 (absence of layer formation) to 3 (layer extending above the lower margin of the pupil). Clot formation (C) is either 0 (no clots in the AC) or 1 (presence of clots). The cumulative total SU-RLC score, therefore, varies from 0 to 7, offering a comprehensive assessment of hyphema severity. The highest total SU-RLC score during postoperative follow-up periods was adopted as the value for each case. In the majority of cases, the highest score was recorded 1–3 days postoperatively.

### 2.3. Surgical Procedures

Details of the surgical procedures have been previously described [3,16,20]. In brief, TMH was preceded by phacoemulsification cataract surgery through a 2.2 mm wide, clear corneal incision at the 9–10 o’clock position (temporal incision for the right eye, nasal incision for the left eye). A one-piece soft acrylic intraocular lens was inserted into the capsular bag through this incision. After cataract surgery, specifically designed spatula-shaped microhooks (M-2215S, 2215R, and 2215L, Inami, Tokyo, Japan) were used for TMH. Viscoelastic material (1% sodium hyaluronate or Opegan Hi) was injected into the anterior chamber through corneal ports created with a 20-gauge microvitreoretinal knife at the 2–3 and 9–10 o’clock positions. A microhook was inserted into the AC through a corneal port, and the angle opposite the port was observed using a Swan–Jacob gonioprism lens. The microhook tip was inserted into Schlemm’s canal and moved circumferentially to incise the inner wall of Schlemm’s canal and TM for more than 3 clock-hour-width at the nasal or both nasal and temporal positions. After TMH, viscoelastic material was aspirated, and corneal ports were closed by corneal stromal hydration. At the end of surgery, a steroid (2 mg of betamethasone sodium phosphate, Rinderone, Shionogi Pharmaceutical) was injected subconjunctivally, and 0.3% ofloxacin ointment (Tarivid, Santen Pharmaceutical) was applied. Postoperatively, 1.5% levofloxacin (Nipro, Osaka, Japan) and 0.1% betamethasone (Sanbetason, Santen Pharmaceutical) were applied topically four times daily for 3–4 weeks.

### 2.4. Statistical Analysis

The measurement parameters were compared between the nasal and bilateral incision groups using the Wilcoxon signed rank test for continuous data, Fisher’s exact probability test for categorical data, and the Cochran–Armitage trend test for ordinal data. Kaplan–Meier curves analyzed the estimated survival probability for qualified IOP control. Successful IOP control was evaluated using survival curve analysis, considering cases that were uncensored when IOP exceeded 15 mmHg (criterion A) or 12 mmHg (criterion B) after 3 months postoperatively, when IOP reduction was less than 20% (both definitions), when additional glaucoma surgery was required at any time (both definitions), or when there was a loss of light perception (both definitions). Cases not meeting these criteria were considered censored. The use or non-use of antiglaucoma medication was not factored into the survival curve analysis, as most cases continued medication postoperatively. Log-rank tests assessed the difference in survival rates between surgical groups. A *p*-value of less than 0.05 was considered statistically significant. All statistical analyses were performed using JMP Pro version 17.1 (SAS Institute, Inc., Cary, NC, USA).

## 3. Results

The demographic data of the subjects, including age, sex, laterality, types of glaucoma, BCVA, IOP, medication score, axial length, AC flare, CECD, and MD values measured preoperatively, are summarized in Table 1. In the nasal and bilateral incision groups, the mean ages were 71.0 years and 71.6 years, respectively, with males constituting 51% and 52% of each group, respectively. There were no significant differences in any of the demographic data between the two procedure groups.

Table 2 presents a comparison of IOP between the nasal and bilateral incision groups. Preoperative IOP, which was 18.6 mmHg in both groups, decreased to 13.2 mmHg (29% reduction) in the nasal incision group and to 12.8 mmHg (31% reduction) in the bilateral incision group at 12 months postoperatively. The IOPs were comparable between the groups throughout the study period of up to 12 months postoperatively.

Table 3 shows the comparison of the number of antiglaucoma medications between the nasal and bilateral incision groups. The preoperative medication score of 3.4 in the nasal incision group and 3.1 in the bilateral incision group was reduced to 2.7 (21% reduction) and 2.5 (19% reduction), respectively, at 12 months postoperatively. The medication scores were similar between groups throughout the study period of up to 12 months postoperatively.

**Figure 1:**
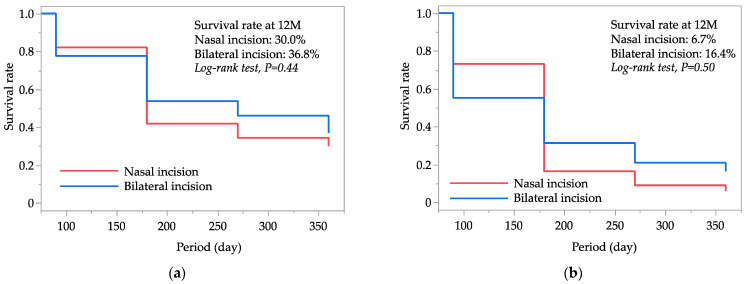
Kaplan–Meier survival curve analyses for successful intraocular pressure (IOP) control in both incision groups by two failure criteria, i.e., criterion A (**a**), IOP reduction < 20% from preoperative IOP and/or >15 mmHg; and criterion B (**b**), IOP reduction < 20% from preoperative IOP and/or >12 mmHg. Patients required additional glaucoma surgery and/or who had no light perception were also classified as failures.

Figure 1 displays the Kaplan–Meier survival curves for successful IOP control in both the nasal and bilateral incision groups. At 12 months postoperatively, the cumulative survival rates for successful IOP control were 30.0% in the nasal incision group and 36.8% in the bilateral incision group based on criterion A, and 6.7% and 16.4%, respectively, based on criterion B. The comparison between the two groups using log-rank statistics yielded *p*-values of 0.44 for criterion A and 0.50 for criterion B, indicating no significant differences in survival rates for successful IOP control between the nasal and bilateral incision groups.

Table 4 presents a comparison of postoperative complications, other than hyphema, and interventions between the nasal and bilateral incision groups. The occurrence of any complications and the need for interventions were not significantly different between the groups. All five additional glaucoma surgeries recorded in this dataset were Ahmed Glaucoma Valve implantations.

Table 5 details the comparison of postoperative hyphema scores between the groups. Postoperatively, the frequency of hyphema, including minimal floating RBCs in the AC, was 97% in the nasal incision group and 100% in the bilateral incision group. The mean total RLC scores were 3.1 in the nasal incision group and 3.5 in the bilateral incision group, which was not statistically significant (*p* = 0.08); the mode of the score was 2 (24%) in the nasal group, while it was 3 (49%) and 4 (34%) in the bilateral incision group. For each component of the hyphema score, score R was significantly higher in the bilateral incision group than in the nasal incision group (*p* < 0.0001), whereas scores L and C were similar between the groups. The incidence of layered hyphema was 33% in the nasal incision group compared to 25% in the bilateral incision group.

Table 6 presents a comparison of BCVAs between the nasal and bilateral incision groups. Preoperative BCVA, which was 0.28 in the nasal incision group and 0.24 in the bilateral incision group, improved to 0.04 in the nasal group and 0.07 in the bilateral group at 12 months postoperatively. The BCVAs were comparable between the groups throughout the study period of up to 12 months.

Table 7 shows the comparison of AC flare between the nasal and bilateral incision groups. The preoperative AC flare was 10.8 pc/ms in both groups, changing to 11.5 pc/ms in the nasal group and 10.6 pc/ms in the bilateral group at 12 months postoperatively. The AC flares were similar between the groups throughout the study period of up to 12 months.

Table 8 details the comparison of CECDs between the nasal and bilateral incision groups. The preoperative CECD was 2468 cells/mm^2^ in the nasal group and 2442 cells/mm^2^ in the bilateral group, decreasing to 2338 cells/mm^2^ (5% reduction) in the nasal group and 2243 cells/mm^2^ (8% reduction) in the bilateral group at 12 months postoperatively. The CECDs were equivalent between the groups throughout the study period of up to 12 months.

Table 9 presents a comparison of visual field MDs between the nasal and bilateral incision groups. The preoperative MD was −14.3 dB in the nasal group and −15.2 dB in the bilateral group, improving to −13.6 dB in the nasal group and −13.4 dB in the bilateral group at 12 months postoperatively. The MDs were equivalent between the groups both preoperatively and at 12 months postoperatively.

## 4. Discussion

In this study of TMH combined with cataract surgery, surgical efficacy indicators such as IOP, medication score, survival rate, BCVA, and visual field MD were comparable between the nasal and bilateral incision groups. Postoperative hyphema was more severe in the bilateral incision group than in the nasal incision group, while other safety profiles, including complications other than hyphema, interventions, AC flare, and CECD, were equivalent between the groups.

A study on perfused autopsy eyes showed that cuts in the trabecular meshwork at width corresponding to 1, 4, and 12 clock hours led to reductions in outflow resistance by 30%, 44%, and 51%, respectively, under a perfusion pressure of 7 mmHg, and by 30%, 56%, and 72%, respectively, under a pressure of 25 mmHg [4]. This suggests that different extents of trabeculotomy can result in varying levels of IOP reduction. Comparing IOP reductions from angle incisions on either one or both sides and exploring the correlation between the extent of these incisions and IOP reductions following goniotomy/trabeculotomy could be insightful. Previous studies, such as those examining the extent of incision after Trabectome (from 0 to 160 degrees) [21] or gonioscopy-assisted transluminal trabeculotomy (GATT) (from 150 to 320 degrees) [8], did not find an association with postoperative IOP. Similarly, a single-center retrospective study observed no significant difference in IOP reduction, medication score, or success rate of IOP reduction between 1-quadrant and 2-quadrants TMH [11]. More recently, a multicenter study reported no significant differences in IOP reduction, medication number, and cumulative survival probability for complete and qualified success rates among 120-, 240-, and 360-degree standalone goniotomy, as well as when these procedures were combined with cataract surgery [13]. In light of these findings, along with the results of our current study, a one-quadrant trabeculotomy may be sufficient for treating most cases of open-angle glaucoma. We found no significant difference in IOP reduction effectiveness across all cases, regardless of the width of the trabecular meshwork incision. However, there might be variability in individual cases, suggesting the need for future research to identify specific case characteristics where a wider trabecular meshwork incision could be more beneficial.

Other studies have reported greater IOP reductions with goniotomy using a Kahook Dual Blade (KDB) at 90 degrees compared to the first-generation iStent trabecular bypass implantation with one 120 µm-lumen stent [22,23,24,25] or the second-generation iStent inject W with two 80 µm-lumen stents [26,27]. In our previous studies, fellow-eye comparisons demonstrated a greater IOP reduction with TMH than with both the first- [28] and second- [29] generation iStent when combined with cataract surgery. Further, other research has shown similar IOP reduction effectiveness between TMH and KDB [30,31]. A recent multicenter study reported comparable IOP reduction effectiveness and safety profiles between one-quadrant TMH and iStent inject W [32]. These findings suggest that goniotomy with a 90-degree incision may achieve a clinically detectable maximal IOP reduction. However, the minimal incision width required to attain maximal IOP reduction to achieve “minimally invasive” surgery warrants further investigation.

We observed that the presence of RBCs in the anterior chamber, as indicated by the SU-R score, was significantly higher in the bilateral incision group than in the nasal incision group. This increase in RBC presence did not correspond with a higher incidence of layered hyphema or blood clots. Postoperative layered hyphema was more frequently reported with a 360-degree incision compared to a 180-degree incision in GATT procedures [9]. Similarly, the incidence of hyphema was more common following goniotomy with a 360-degree incision than with a 120-degree incision [12,13]. In our previous research, we reported higher hyphema scores in eyes undergoing TMH than in those receiving first- and second-generation iStent implants during the early postoperative period [19,29]. These findings suggest that the severity of hyphema correlates with the extent of incision width following angle surgeries. However, the differences in SU-R scores during the early postoperative period did not impact other safety profiles or the final visual function, although they likely influenced the early recovery of vision. Therefore, if the effectiveness of IOP reduction is similar between the two groups, the nasal-incision approach, which may permit a faster recovery of visual function, could be considered clinically more advantageous than the bilateral-incision method.

The study had several limitations, including its retrospective design and lack of randomization. Propensity score matching was utilized to minimize these limitations. The study primarily included cases treated with combined TMH and cataract surgery, predominantly involving an elderly population, which may limit the generalizability of the findings. Lack of information on the use of anticoagulants and antiplatelet agents was also a study limitation. The relatively short follow-up period is another limitation. However, the study also had strengths, such as a comprehensive assessment of patient clinical characteristics and a matched-group analysis to mitigate the influence of background factors.

## 5. Conclusions

In conclusion, our study demonstrated that the extent of IOP and medication number reductions achieved with TMH combined with cataract surgery were equivalent, irrespective of incision width. While the overall safety profiles were similar, procedures with a narrower incision width resulted in a reduced presence of AC RBCs in the early postoperative period.

## Figures and Tables

**Table 1:** Demographic subject data.

Parameters	Nasal Incision	Bilateral Incision	*p*-Value
N, eyes	67	67	
Age, years			
Mean ± SD	71.0 ± 8.1	71.6 ± 0.9	0.48
95% CI	69.2, 72.7	70.0, 73.2	
Sex, *n* (%)			
Male	34 (51)	37 (52)	1.0
Female	33 (49)	32 (48)	
Laterarity, *n* (%)			
Right eye	30 (45)	23 (34)	0.29
Left eye	37 (55)	44 (66)	
Glaucoma type, *n* (%)			
POAG	45 (67)	45 (67)	1.0
EXG	22 (33)	22 (33)	
BCVA, LogMAR			
Mean ± SD	0.28 ± 0.5	0.24 ± 0.5	0.33
95% CI	0.16, 0.39	0.12, 0.35	
IOP, mmHg			
Mean ± SD	18.6 ± 5.2	18.6 ± 5.1	0.94
95% CI	17.3, 19.9	17.3, 19.8	
Medication score			
Mean ± SD	3.4 ± 0.8	3.1 ± 1.2	0.49
95% CI	3.2, 3.6	2.9, 3.4	
Axial length, mm			
Mean ± SD	24.7 ± 1.4	24.8 ± 1.7	0.83
95% CI	24.3, 25.0	24.4, 25.2	
AC flare, pc/ms			
Mean ± SD	10.8 ± 6.1	10.8 ± 12.2	0.13
95% CI	9.3, 12.3	10.4, 14.7	
CECD, cells/mm^2^			
Mean ± SD	2468 ± 266	2442 ± 302	0.98
95% CI	2403, 2533	2369, 2516	
MD, dB			
Mean ± SD	−14.3 ± 8.7	−15.2 ± 8.2	0.64
95% CI	−16.5, −12.2	−17.2, −13.2	

*p* values are calculated by Wilcoxon signed rank test for continuous data and using Fisher’s exact probability test for categorical data. SD, standard deviation; CI, confidence interval; POAG, primary open-angle glaucoma; EXG, exfoliation glaucoma; BCVA, best-corrected visual acuity; IOP, intraocular pressure; AC, anterior chamber; pc/ms, photon counts per milli-second; CECD, corneal endothelial cell density; MD, visual field mean deviation; dB, decibels.

**Table 2:** Pre- and post-operative IOPs.

Parameters	Nasal Incision	Bilateral Incision	*p*-Value
Preoperative			
Mean ± SD, mmHg	18.6 ± 5.2	18.6 ± 5.1	0.94
95% CI	17.3, 19.9	17.3, 19.8	
POD3			
Mean ± SD	12.7 ± 8.0	12.4 ± 7.7	0.73
95% CI	10.7, 14.6	10.6, 14.3	
POW2			
Mean ± SD	14.8 ± 4.3	14.0 ± 5.1	0.10
95% CI	13.7, 15.9	12.7, 15.3	
POM1			
Mean ± SD	13.3 ± 3.4	13.0 ± 4.8	0.46
95% CI	12.4, 14.2	11.3, 14.7	
POM3			
Mean ± SD	13.1 ± 2.8	12.7 ± 3.3	0.49
95% CI	12.0, 14.1	11.8, 13.6	
POM6			
Mean ± SD	13.9 ± 3.2	12.9 ± 3.6	0.08
95% CI	13.1, 14.6	12.0, 13.8	
POM9			
Mean ± SD	13.9 ± 4.5	12.7 ± 2.7	0.21
95% CI	12.7, 15.0	12.0, 13.4	
POM12			
Mean ± SD	13.2 ± 3.5	12.8 ± 2.9	0.80
95% CI	12.3, 14.1	12.1 ± 13.6	

*p* values are calculated by Wilcoxon signed rank test. SD, standard deviation; CI, confidence interval; POD, postoperative day; POW, postoperative week; POM, postoperative month.

**Table 3:** Pre- and post-operative medication score.

Parameters	Nasal Incision	Bilateral Incision	*p*-Value
Preoperative			
Mean ± SD	3.4 ± 0.8	3.1 ± 1.2	0.49
95% CI	3.2, 3.6	2.9, 3.4	
POD3			
Mean ± SD	2.1 ± 0.8	2.3 ± 0.8	0.13
95% CI	1.9, 2.3	2.1, 2.5	
POW2			
Mean ± SD	2.1 ± 0.8	2.2 ± 0.8	0.31
95% CI	1.9, 2.3	2.0, 2.4	
POM1			
Mean ± SD	2.1 ± 0.8	2.2 ± 0.8	0.48
95% CI	1.9, 2.4	1.9, 2.5	
POM3			
Mean ± SD	2.3 ± 0.9	2.3 ± 0.8	0.82
95% CI	2.0, 2.7	2.0, 2.5	
POM6			
Mean ± SD	2.5 ± 0.9	2.4 ± 0.8	0.70
95% CI	2.2, 2.7	2.2, 2.6	
POM9			
Mean ± SD	2.7 ± 0.9	2.5 ± 0.8	0.33
95% CI	2.4, 2.9	2.3, 2.7	
POM12			
Mean ± SD	2.7 ± 0.9	2.5 ± 0.9	0.27
95% CI	2.4, 2.9	2.3, 2.7	

*p* values are calculated by Wilcoxon signed rank test. SD, standard deviation; CI, confidence interval; POD, postoperative day; POW, postoperative week; POM, postoperative month.

**Table 4:** Postoperative complications (except for hyphema) and interventions.

Parameters	Nasal Incision	Bilateral Incision	*p*-Value
Fibrin formation	3 (4)	6 (9)	0.49
IOP spike (>30 mmHg)	4 (6)	1 (1)	0.37
Additional glaucoma surgery	4 (6)	1 (1)	0.37
Hyphema washout	1 (1)	3 (4)	0.62
YAG capsulotomy	3 (4)	0 (0)	0.24
tPA fibrinosis	1 (1)	0 (0)	1.0
Psoterior syechialysis	0 (0)	1 (1)	1.0

*p* values are calculated by Wilcoxon signed rank test. IOP, intraocular pressure; YAG, Yttrium–Aluminum–Garnet laser; tPA, tissue plasminogen activator.

**Table 5:** Postoperative hyphema severity scored by SU-RLC scoring system.

Parameters	Nasal Incision	Bilateral Incision	*p*-Value
Any hyphema, n (%)	65 (97)	67 (100)	0.50
Total RLC score			
Mean ± SD	3.1 ± 1.5	3.5 ± 0.9	0.08
95% CI	2.7, 3.5	3.3, 3.7	
0, *n* (%)	2 (3)	0 (0)	0.08
1	7 (10)	2 (3)	
2	16 (24)	2 (3)	
3	15 (22)	33 (49)	
4	15 (22)	23 (34)	
5	8 (12)	6 (9)	
6	4 (6)	1 (1)	
7	0 (0)	0 (0)	
R score			
0, *n* (%)	2 (3)	0 (0)	<0.0001
1	13 (19)	2 (3)	
2	16 (24)	9 (13)	
3	36 (54)	56 (84)	
L score			
0, *n* (%)	45 (67)	50 (75)	0.57
1	18 (27)	12 (18)	
2	4 (6)	5 (7)	
3	0 (0)	0 (0)	
C score			
0, *n* (%)	38 (57)	44 (66)	0.29
1	29 (43)	23 (34)	

*p* values are calculated by Wilcoxon signed rank test for continuous data and using Cochran–Armitage trend test for ordinal data. SU-RLC, Shimane University postoperative hyphema scoring system; R-score, scores for floating red blood cell density in anterior chamber; L score, scores for layered hyphema; C score, scores for blood clot in anterior chamber; SD, standard deviation; CI, confidence interval.

**Table 6:** Pre- and post-operative BCVA.

Parameters	Nasal Incision	Bilateral Incision	*p*-Value
Preoperative			
Mean ± SD, LogMAR	0.28 ± 0.5	0.24 ± 0.5	0.33
95% CI	0.16, 0.39	0.12, 0.35	
POW2			
Mean ± SD	0.25 ± 0.43	0.23 ± 0.48	0.59
95% CI	0.14, 0.35	0.11, 0.35	
POM1			
Mean ± SD	0.12 ± 0.26	0.17 ± 0.47	0.86
95% CI	0.05, 0.18	0.01, 0.34	
POM3			
Mean ± SD	0.15 ± 0.35	0.08 ± 0.39	0.19
95% CI	0.01, 0.28	−0.03, 0.18	
POM6			
Mean ± SD	0.08 ± 0.25	0.11 ± 0.48	0.20
95% CI	0.02, 0.14	−0.01, 0.23	
POM9			
Mean ± SD	0.04 ± 0.20	0.09 ± 0.48	0.80
95% CI	−0.01, 0.09	−0.04, 0.21	
POM12			
Mean ± SD	0.04 ± 0.19	0.07 ± 0.42	0.31
95% CI	−0.00, 0.09	−0.04, 0.17	

*p* values are calculated by Wilcoxon signed rank test. BCVA, best-corrected visual acuity; LogMAR, logarithm of the minimum angle of resolution; SD, standard deviation; CI, confidence interval; POW, postoperative week; POM, postoperative month.

**Table 7:** Pre- and post-operative AC flare.

Parameters	Nasal Incision	Bilateral Incision	*p*-Value
Preoperative			
Mean ± SD, pc/ms	10.8 ± 6.1	10.8 ± 12.2	0.13
95% CI	9.3, 12.3	10.4, 14.7	
POW2			
Mean ± SD	38.7 ± 25.7	36.9 ± 23.9	0.80
95% CI	32.3, 45.0	30.9, 42.8	
POM1			
Mean ± SD	24.5 ± 14.9	21.6 ± 14.5	0.23
95% CI	20.5, 28.5	16.3, 26.8	
POM3			
Mean ± SD	18.5 ± 10.1	16.2 ± 7.6	0.41
95% CI	14.7, 22.3	14.0, 18.4	
POM6			
Mean ± SD	12.9 ± 6.5	12.7 ± 6.0	0.85
95% CI	11.2, 14.5	11.2, 14.2	
POM9			
Mean ± SD	12.6 ± 5.7	11.2 ± 4.7	0.16
95% CI	11.2, 14.0	10.0, 12.3	
POM12			
Mean ± SD	11.5 ± 5.9	10.6 ± 4.6	0.73
95% CI	10.0, 13.0	9.4, 11.7	

*p* values are calculated by Wilcoxon signed rank test. AC, anterior chamber; pc/ms, photon counts per milli-second; SD, standard deviation; CI, confidence interval; POW, postoperative week; POM, postoperative month.

**Table 8:** Pre- and post-operative CECD.

Parameters	Nasal Incision	Bilateral Incision	*p*-Value
Preoperative			
Mean ± SD, cells/mm^2^	2468 ± 266	2442 ± 302	0.98
95% CI	2403, 2533	2369, 2516	
POM6			
Mean ± SD	2313 ± 287	2233 ± 292	0.23
95% CI	2240, 2387	2161, 2307	
POM12			
Mean ± SD	2338 ± 262	2243 ± 334	0.18
95% CI	2272, 2404	2161, 2326	

*p* values are calculated by Wilcoxon signed rank test. CECD, corneal endothelial cell density; SD, standard deviation; CI, confidence interval; POM, postoperative month.

**Table 9:** Pre- and post-operative visual field MD.

Parameters	Nasal Incision	Bilateral Incision	*p*-Value
Preoperative			
Mean ± SD, dB	−14.3 ± 8.7	−15.2 ± 8.2	0.64
95% CI	−16.5, −12.2	−17.2, −13.2	
POM12			
Mean ± SD	−13.6 ± 8.8	−13.4 ± 9.1	0.73
95% CI	−15.8, −11.4	−15.7, −11.1	

*p* values are calculated by Wilcoxon signed rank test. MD, mean deviation; dB, decibel; SD, standard deviation; CI, confidence interval; POM, postoperative month.

## Data Availability

Data are fully available upon reasonable request to corresponding author.
